# Effect of Using an 8-Figure Shoulder Brace on Posture and Muscle Activities during the Performance of Dental Hygiene Procedures

**DOI:** 10.3390/ijerph17228494

**Published:** 2020-11-16

**Authors:** Tae-Lim Yoon, Ji-Hyun Min, Han-Na Kim

**Affiliations:** 1 Department of Physical Therapy, College of Health and Medical Sciences, Cheongju University, Cheongju 28503, Korea; taelimyoon@cju.ac.kr; 2 Department of Dental Hygiene, College of Health and Medical Sciences, Cheongju University, Cheongju 28503, Korea; jhmin@cju.ac.kr

**Keywords:** dental hygiene, dental scaling, EMG activity, musculoskeletal disorder, 8-figure shoulder brace

## Abstract

The incidence of work-related musculoskeletal disorders (MSDs) among dental workers has been increasing. Many ergonomic devices and accessories have been introduced. The aim of this study was to investigate the effects of an 8-figure shoulder brace on posture-related muscle activities in dental hygiene practitioners during scaling procedures. In this study, 33 participants (age: 21.9 ± 2.1 years, height: 162.0 ± 6.0 cm, weight: 55.8 ± 9.0 kg, body mass index: 21.2 ± 2.4 kg/m^2^) performed the scaling procedure with and without the 8-figure shoulder brace in a randomized order. The normalized electromyography activity in the amplitude probability distribution function and joint angles (cervical, thoracic, lumbar, and shoulder joints) were simultaneously recorded during scaling. A paired *t* test was used to compare the differences in muscle kinematics, with the alpha level set at 0.05. The dental hygienists who wore the 8-figure shoulder brace during scaling showed thoracic and lumbar extension, improved sitting postures, and reduced shoulder joint abduction. However, we also observed an unintended increase in internal rotation. Use of the 8-figure shoulder brace could prevent work-related MSDs in lumbar and thoracic regions by reducing the effort exerted by the upper trapezius and deltoid muscles, despite the increased muscular effort of the cervical erector spinae.

## 1. Introduction

Dental workers such as dentists and dental hygienists repeatedly overstress their arms and hands for a long time in an awkward position to treat small teeth in the oral cavity. As a result, they often have work-related musculoskeletal disorders (MSDs). According to recent studies, 64–93% of dental workers complain of musculoskeletal pain, primarily in the neck, shoulder, wrist, and upper back [1,2]. According to another study, back pain was the most frequent problem reported by one of four dental workers, followed by neck and shoulder pain [3].

Scaling, one of the representative tasks of dental hygienists, is a dental hygiene procedure that involves removal of dental plaques and calculus. In this task, excessive stress is applied to the hands and arms, and the small muscles of the fingers are repeatedly used, which can cause MSDs [3,4,5]. According to previous studies, use of excessive force, repetitive task, and the work environment are risk factors of MSDs, and scaling is a task that is highly related to these risk factors [3,6]. The awkward posture related to work increases the stress on the neck, shoulder, and back muscles, which leads to shoulder rotator cuff syndrome, shoulder tendinitis, neck pain, and low back pain [4]. As a result, MSDs decrease dental workers’ productivity and consequently reduce their earnings. Therefore, measures must be taken to address the occurrence of MSDs in dental workers.

Several ergonomic rules are recommended to prevent work-related musculoskeletal pain in dental workers. According to one study, proper body posture and position while performing dental procedures significantly reduced the risk of work-related musculoskeletal pain [6]. When performing a dental procedure, the correct posture for the dental worker includes adjusting the dental chair to fit the operator’s body shape, aligning the torso to match the long axis of the body, aligning the height of both shoulders side by side, and positioning the operator’s elbows close to the side of the torso [7,8]. In addition, strategies such as regular stretch breaks, changing operator and client chair positions, use of magnification telescopes, regular physical exercise such as yoga, and increased operator awareness regarding MSDs effectively prevented the occurrence of MSDs in dental workers [6]. Other researchers suggested that the use of loupes can reduce neck pain and disability among dental hygienists [9].

Braces are conventionally used to improve body posture and prevent work-related MSDs. Recently, an 8-figure shoulder brace with an elastic strap was recommended to correct neck and shoulder malpositions in the standing posture, and prevent occupational musculoskeletal problems. Studies have demonstrated that the strategy of using a brace or scapular posterior tilt exercise effectively corrects cervical hyperlordosis, round shoulder, thoracic kyphosis, and lumbar lordosis [4,10]. Other previous studies confirmed that it produced positive changes in the musculoskeletal system of desk workers and overhead athletes. To date, no study has been conducted to evaluate the performance of the shoulder brace during dental procedures. The aim of this study was to investigate the effect of the 8-figure shoulder brace on posture and related muscle activities during the performance of dental hygiene procedures.

## 2. Materials and Methods

### 2.1. Study Participants

Thirty-three healthy subjects with at least 2 years of experience in manual scaling (32 women and 1 man; mean age, 21.9 ± 2.1 years; height, 162.0 ± 6.0 cm; weight, 55.8 ± 9.0 kg; body mass index, 21.2 ± 2.4 kg/m^2^; and hand dominance, right 30/left 3) participated in the study. The exclusion criteria were cervical pathologies, psychiatric conditions, neurological or MSDs, previous neck surgeries, and recent musculoskeletal trauma affecting the neck range of motion. The population included in the study was a convenience sample recruited by purposive and snowball sampling. The study was approved by the Cheongju University Institutional Review Board for Human Subject Research (1041107-161228-HR-008-04). We obtained written informed consent from all the subjects who participated in the study.

### 2.2. Instruments

#### 2.2.1. Three-Dimensional Motion Analysis

A three-dimensional (3-D) motion analysis system using inertial measurement units (IMUs) was used to analyze the effect of the 8-figure shoulder brace on posture while performing dental hygiene procedures. IMUs offer a low-cost and easy-to-use alternative to optoelectronic measurement systems for measuring 3-D body motions [11]. Nine inertial sensors were attached to the subject (head, C7, pelvis, right and left upper arm, right and left lower arms, and right and left dorsal parts of the hand) with special straps to capture the motion data with the MyoMotion system (Noraxon, Inc., Scottsdale, AZ, USA). The inertial sensor unit consisted of acceleration sensors, three gyroscope sensors, and one magnetic sensor to calculate 3-D coordinates and joint angles at a sampling frequency of 200 Hz. The joint angles were measured at the neck, shoulder, and waist joints. As the measurement was 3-D, all 3-D angular ranges could be measured. The MyoMotion system was calibrated once for each participant in a sitting position, with a flexed arm and defined corrective posture. All the body motion data were analyzed using the user interface MR3 [12].

#### 2.2.2. Electromyography Recording and Data Processing

Surface electromyography (EMG; Telemyo 2400T, Noraxon Co, Scottsdale, AZ, USA) was used to measure the muscle activities of the cervical erector spinae (CES), upper trapezius (UT), deltoid, and thoracic erector spinae (TES) of the subjects. The measured values were calculated using the Noraxon MR 3.8 software (Noraxon, Scottsdale, AZ, USA) installed in the measuring equipment. Before attaching the electrodes, all the parts were cleaned with alcohol and thoroughly dried to minimize skin resistance. The electrode for measuring muscle activity was attached perpendicular to the contralateral side of the CES, UT, deltoid, and TES muscles, keeping a distance of 2 cm between the electrodes. The surface of the electrode was attached to the CES, UT, deltoid, and TES muscles bilaterally as described in a previous study [6]. The maximal voluntary isometric contraction was evaluated three times in the CES, UT, deltoid, and TES muscles, as recommended by Kendall et al. [13]. The sampling rate was set at 1500 Hz; the notch filter, at 60 Hz; and the band-pass filter, at 20–450 Hz.

Normalized EMG data were processed to estimate the amplitude probability distribution function (APDF). The APDF has been extensively used in ergonomic research to suggest risk thresholds for occupational exposures at specific muscle activity amplitudes [14,15,16]. The 10th, 50th, and 90th percentile APDFs represent low, medium, and high levels of muscle activity, respectively [7,17]. The 10th percentile is an indicator of static or low amplitude postural muscle movement. The 50th percentile is an indicator of intermediate or moderate muscular effort, and the 90th percentile indicates the peak or high-level muscle activity amplitude [18,19].

#### 2.2.3. Application of the 8-Figure Shoulder Brace

**Figure 1:**
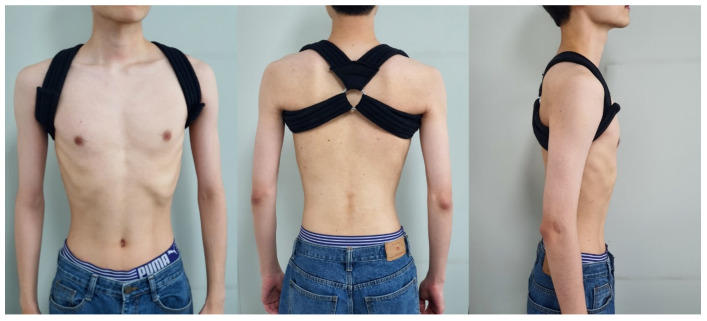
The 8-figure shoulder brace.

A previous study reported that the 8-figure shoulder brace modified the scapular alignment and muscle activity of subjects with rounded shoulder postures [4]. The principal investigator (YTL) used the 8-figure shoulder brace (Theplus, Inc., Seoul, Korea) in accordance with the manufacturer’s specifications, using a 1-strap method to adjust the strap tension (Figure 1) [4]. The subjects sat with both arms raised while wearing the shoulder brace. The investigator wrapped the strap at the subject’s coracoid process on the right shoulder first. He pulled the strap from the mid-thorax, crossed the coracoid process and axillary area, and returned back at the mid-thorax. The same procedure was repeated on the left side.

### 2.3. Procedure

**Figure 2:**
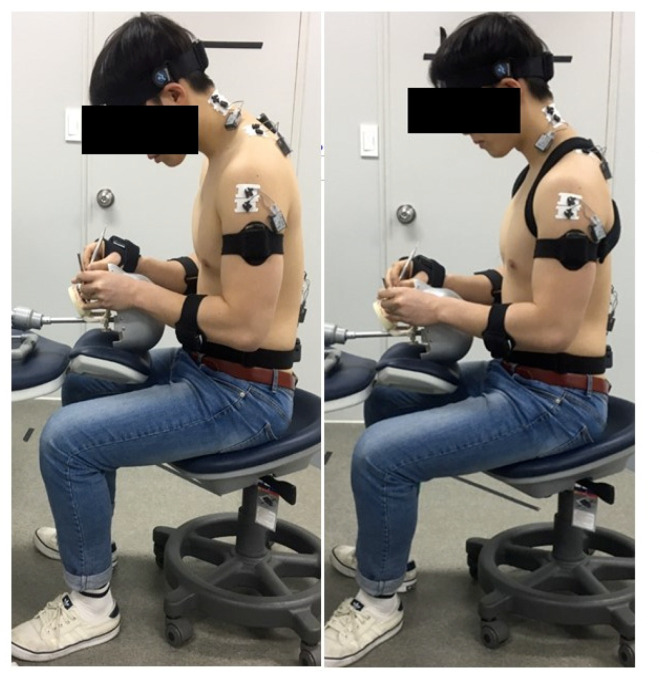
Manual scaling task with and without applying the 8-figure shoulder brace.

The subjects sat on swivel stools without backrests and performed scaling on dental manikins (Nissim Type 2, Nissin Dental Products Inc., Kyoto, Japan). The manikins were mounted as close to the dental field as possible. Prior to data collection, the subjects were familiarized with the procedure to ensure accurate results. The teeth used in the study were the lateral incisors (distal surfaces of Nos. 12, 22, 32, and 42) placed in a typodont. Each tooth received a prior coat of nail varnish to simulate plaque and calculus deposits. The principal investigator trained each subject to perform the simulated manual scaling. The subjects had to remove the nail varnish with a Gracey 1/2 curette (SG1/26, Hu-Friedy Mfg. Co., Chicago, IL, USA) or anterior sickle scaler (H5/33, Hu-Friedy Mfg. Co., Chicago, IL, USA) for as long as possible, without damaging the tooth structure (Figure 2). The subjects could change the position of the chair between the 7 and 12 o’clock positions, depending on the area under operation. The EMG activity and joint angles (cervical, thoracic, lumbar, and shoulder joints) were simultaneously recorded during scaling. The scaling took approximately 2 min to complete for each tooth surface and 8 min for the four lateral incisor teeth. The order of scaling with and without the application of the 8-figure shoulder brace was randomized. To avoid muscle fatigue, the subjects were allowed to rest for 5 min while changing the instruments [20].

### 2.4. Statistical Analyses

The normal distribution of all the acquired data was confirmed with the Kolmogorov-Smirnov test. A paired *t* test was used to compare the differences in muscle kinematics and kinetic variables before and after using the 8-figure shoulder brace during scaling. All the analyses were performed using the SPSS version 24.0 statistical software (IBM Corp., New York, NY, USA), and the alpha level was set at 0.05.

## 3. Results

**Figure 3:**
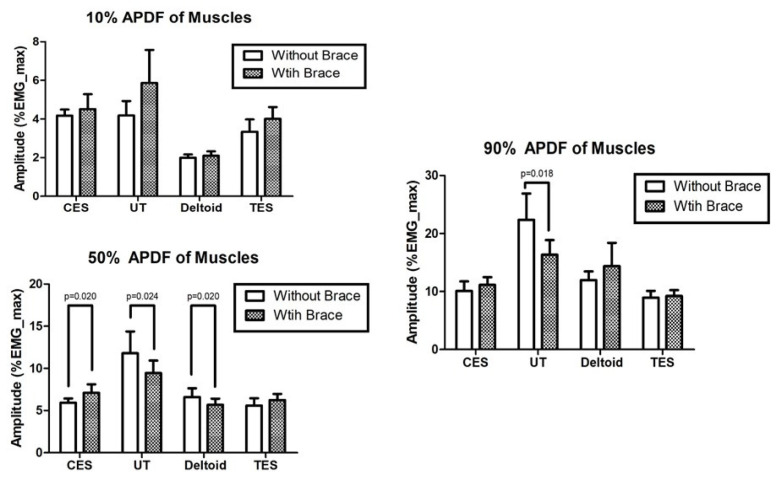
Muscle activity levels with and without the 8-figure shoulder brace; EMG, Surface electromyography; CES, Cervical erector spinae; UT, Upper trapezius; TES, thoracic erector spinae; APDF, Amplitude probability distribution function.

Significant differences were found in thoracic flexion, lumbar flexion and side bending, right shoulder rotation, and left shoulder abduction (*p* < 0.05; Table 1). With the use of the brace, the 50% APDF of the CES significantly decreased, while the 50% APDF of the deltoid increased. The 50% and 90% APDFs of the UT significantly decreased with the use of the brace (*p* < 0.05). We found no significant change in the 90% APDF of the UT (Figure 3).

## 4. Discussion

The aim of the study was to investigate the effects of an 8-figure shoulder brace on posture-related muscle activities in dental hygiene practitioners during scaling. The results of the study demonstrated a change in body posture and muscle activity.

The subjects who used the 8-figure shoulder brace showed a significantly reduced thoracic flexion (approximately 4.3°). In some studies, the use of the 8-figure shoulder brace showed an immediate effect on spinal and scapular alignments, with an average correction of 36% of the initial value [21,22]. Thoracic kyphosis usually pulls the body to a forward bending position that is associated with decreased physical function, decreased respiratory function, poor postural control, and poor quality of life [20,21]. The scaling procedure performed by the dental hygienists involves prolonged sitting and increases the risk of thoracic kyphosis [23]. Dental hygienists who use the 8-figure shoulder brace during scaling could prevent the worsening of thoracic kyphosis.

Although the use of the brace showed a predictable, immediate reduction in thoracic flexion, an unexpected significant increase in lumbar extension (approximately 0.6°) and a decrease in side bending (approximately 1.8°) were observed. Several studies have reported changes in the lumbar angles when thoracic kyphosis changed [24,25]. As the previous studies that reported reductions in thoracic kyphosis and lumbar lordosis with the use of a shoulder brace were conducted in a standing position, different results may be obtained when the brace is used in a sitting position as in our study. A study that compared slumped sitting and sitting straight showed that the thoracic and lumbar extension angles increased during upright sitting [26]. This study suggested that the 8-figure shoulder brace affected the alignments of both the thoracic and lumbar spines. Thus, we assumed that the reduction in thoracic flexion by the effect of the 8-figure shoulder braces was associated with the lumbar extension as in the straight sitting posture. Several studies reported that a straight sitting posture reduces neck- and shoulder-related musculoskeletal problems [27,28]. The use of an 8-figure shoulder brace enables an upright posture that is beneficial in most workplaces.

We observed that the 8-figure shoulder brace decreased left shoulder abduction (from 16.0° to 9.4°) and increased shoulder internal rotation (from 20.9° to 44.9°) in the right arm during scaling. We assume that the upright posture favorably altered the alignments of the lumbar and thoracic spines, which led to angular changes of the arms. According to a previous study, prolonged upper arm abduction was one of the risk factors for musculoskeletal problems of the shoulders [29]. Hence, even a slight decrease in shoulder abduction may have a positive effect on the deltoid and supraspinatus abductor muscles. Our study showed an increased shoulder internal rotation, a known risk factor for shoulder-related MSDs such as supraspinatus or biceps tendinitis, glenohumeral instability, impingement, and pain [30,31]. However, it can be said that light manual work involving <45° shoulder rotation is less risky than work with heavy loads [32]. The 8-figure shoulder brace increases the shoulder internal rotation, but not by >45°. Hence, we believe that it may not lead to musculoskeletal injuries in the operator. As far as its effect on the shoulder joint is concerned, the 8-figure shoulder brace has both advantages and limitations. However, it is unlikely to have a significant effect on the musculoskeletal system.

Our results on muscle activities showed that the median level increased in the CES and decreased in the UT and deltoid with the use of the 8-figure shoulder brace. Previous studies reported that excessive tonic activation of the neck extensor (CES and UT) and scapular stabilizer (LT) muscles during the performance of dental procedures is related to MSDs [29]. The 8-figure brace had the advantage of reducing the middle-level muscle activities of the UT and deltoid, and the disadvantage of increasing the middle-level muscle activity of the CES. Our results confirmed that the peak muscle activity level of the UT was reduced significantly. Although the median-level muscle activities of the CES and trapezius are reportedly important, high-level muscle activity is also an important measure of muscular effort related to MSDs [21]. The use of the 8-figure shoulder brace could prevent MSDs in dental hygienists by reducing the muscular effort of the UT and deltoid muscles. For a dental hygienist to perform productive tasks with a healthy body, a good sitting posture in the neutral spine position could maintain the curve of the spine naturally when performing dental procedures.

Our study had several limitations. As most of the subjects were young women, the results can only be generalized to this population. Another limitation was that the subjects performed only partial manual scaling, not a full-mouth prophylaxis. Further studies are needed to understand how the 8-figure shoulder brace affects the performance of dental hygienists. Moreover, longitudinal studies on the effect of the brace on MSDs are necessary.

## 5. Conclusions

The dental hygienists who wore the 8-figure shoulder brace while scaling showed thoracic and lumbar extension and an improved sitting posture that prevented the slumped sitting posture-related to MSDs. Wearing the brace had the advantage of reduced shoulder joint abduction but a disadvantage of increased shoulder internal rotation. In conclusion, the use of the 8-figure shoulder brace can reduce the awkward working postures leading to MSDs by reducing the effort of the UT and deltoid muscles, although it does increase the muscular effort of the CES.

## Figures and Tables

**Table 1:** Three-dimensional motion analysis with and without the 8-figure shoulder brace.

	Without Brace (Mean ± SD)	With Brace (Mean ± SD)	95% Confidence Interval	*p* Value
Cervical flexion (+)/extension (−)	21.7 ± 11.3	24.7 ± 12.0	−6.5 to 0.4	0.079
Cervical right (+)/left (−) side bending	16.4 ± 9.9	18.1 ± 12.3	−4.5 to 1.1	0.234
Cervical rotation	−1.9 ± 6.5	−1.4 ± 5.1	−2.9 to 2.0	0.706
Thoracic flexion	16.9 ± 9.7	12.6 ± 9.3	1.7–7.0	0.027
Thoracic side bending	7.4 ± 6.5	3.3 ± 6.3	2.2–6.0	0.065
Thoracic rotation	−4.6 ± 7.0	−4.6 ± 6.1	−2.5 to 2.5	0.721
Lumbar flexion	0.3 ± 5.7	−0.3 ± 5.5	−1.3 to 2.5	0.002
Lumbar side bending	−2.9 ± 6.1	−1.1 ± 6.1	−3.6 to 0.1	0.000
Lumbar rotation	−0.3 ± 4.4	−4.6 ± 6.1	1.3–7.3	0.990
Right shoulder flexion (+)/extension (−)	27.7 ± 13.4	24.5 ± 11.1	−1.7 to 8.0	0.889
Right shoulder abduction (+)/adduction (−)	33.6 ± 14.8	31.1 ± 13.6	−2.9 to 8.0	0.195
Right shoulder external (+)/internal (−) rotation	−20.9 ± 17.0	−44.9 ± 18.3	−3.7 to 9.2	0.000
Left shoulder flexion (+)/extension (−)	22.4 ± 9.1	22.2 ± 8.3	−2.3 to 2.7	0.350
Left shoulder abduction (+)/adduction (−)	−16.0 ± 9.9	−9.4 ± 8.6	−8.9 to 4.3	0.025
Left shoulder external (+)/internal (−) rotation	−19.3 ± 9.5	−24.2 ± 13.6	0.6–9.1	0.264
